# Immediate postoperative tracheal extubation in a liver transplant recipient with encephalopathy and the Mayo end-stage liver disease score of 41: A CARE-compliant case report revealed meaningful challenge in recovery after surgery (ERAS) for liver transplantation

**DOI:** 10.1097/MD.0000000000008467

**Published:** 2017-11-27

**Authors:** Jianbo Li, Chengdi Wang, Nan Chen, Jiulin Song, Yan Sun, Qin Yao, Lunan Yan, Jiayin Yang

**Affiliations:** a Department of Liver Surgery and State Key Laboratory of Biotherapy, West China Hospital, Sichuan University; b Department of Respiratory and Critical Care Medicine, West China Medical School/West China Hospital; c West China School of Medicine/West China Hospital, Sichuan University; d Department of Anesthesiology, West China Hospital of Sichuan University, Chengdu, Sichuan, P.R. China.

**Keywords:** enhanced recovery after surgery, immediate postoperative tracheal extubation, liver transplantation, Mayo end-stage liver disease

## Abstract

**Rationale:**

Immediate postoperative tracheal extubation (IPTE) is one of the most important subject in recovery after surgery (ERAS) for liver transplantation. However, the criteria for IPTE is not uniform at present.

**Patient concerns:**

We reported a successful IPTE in a liver transplant recipient with encephalopathy and a high Mayo end-stage liver disease (MELD) score of 41, which beyond the so-called criteria reported in the literature. The patient was 48-year-old man, admitted in September 2016 for end-stage liver cirrhosis secondary to hepatitis B.

**Diagnoses:**

End-stage liver cirrhosis secondary to hepatitis B with encephalopathy and a high MELD score of 41.

**Interventions:**

He was involved in our ERAS project and was extubated at the end of the liver transplantation in the operating room.

**Outcomes:**

As a result, the patient was not reintubated and had an excellent postoperative recovery, staying in intensive care unit (ICU) for just 2 days and discharged home on day 10.

**Lessons:**

We believed IPTE in liver transplant recipients with severe liver dysfunction is a meaningful challenge in ERAS for liver transplantation. Our case and literature review suggest 3 things: IPTE in liver transplantation is generally feasible and safe; the encephalopathy or high MELD score should not be the only limiting factor; and a more systematic predicting system for IPTE in liver transplantation should be addressed in future studies.

## 1 Introduction

Immediate postoperative tracheal extubation (IPTE) as a potential subtopic of the up-to-date concept of enhanced recovery after surgery (ERAS) has been identified as an excellent tool to achieve rapid recovery for selective patients undergoing liver transplantation. However, the criteria for IPTE have not yet been determined. The Mayo end-stage liver disease (MELD) score of <11 is the earliest proposed criteria^[1]^ and the encephalopathy is usually regarded as exclude status for IPTE.^[2–4]^ In this report, we present a successful IPTE in a liver transplant recipient with encephalopathy and a high MELD score up to 41. We also reviewed the relevant literature with a view to the criteria for IPTE and investigated another 6 liver transplantations in the same month in our center with the purpose of identifying potential factors of IPTE in our cases.

## 2 Case report

A 48-year-old man was admitted in September 2016 for end-stage liver cirrhosis secondary to hepatitis B. The patient's decompensated features included marked ascites, esophageal varices, and hepatic encephalopathy. His medical history included an artificial liver support for 10 times in the past month, and type 2 diabetes with bad blood glucose control for 8 years. The full-size liver transplant from a voluntary deceased donor who died of stroke was performed on the day of admission. IPTE was carried out at the end of surgery in the operating room and no complication occurred during the postoperative period. Written informed consent was obtained from each patient and the study application was approved by the ethics committee of West China Hospital.

**Figure 1:**
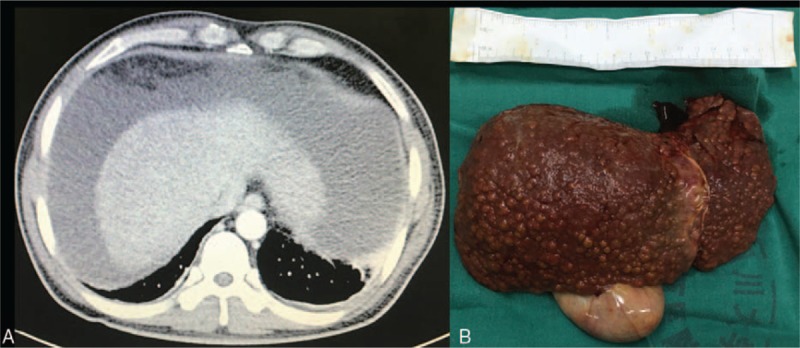
Preoperative contrast-enhanced abdominal computed tomography scan and the resected liver of recipient. (A) Computed tomography enhanced scan of the upper abdomen showed massive ascites and atrophic liver, (B) the gross multinodular cirrhosis appearance of the explanted liver.

Preoperative laboratory investigation showed a bilirubin of 31.2 mg/dL (upper limit of normal reference range 1.6 mg/dL), albumin of 35.6 g/L (lower limit of normal reference range 40 g/L), creatinine of 1.41 mg/dL, prothrombin time of 64.1 seconds (upper limit of normal reference range 12.8 seconds), international standardized ratio of 5.01 (upper limit of normal reference range 1.15), blood ammonia of 81.0 μmol/L (upper limit of normal reference range 33.0 μmol/L), hepatitis B virus DNA of 1.65E + 03 IU/mL (upper limit of normal reference range 1.00E + 02 IU/mL), sodium of 142.6 mmol/L, potassium of 3.96 mmol/L, white cell count of 7.3 × 10^9^/L, lymphocyte count of 1.0 × 10^9^/L, hemoglobin of 99 g/L (lower limit of normal reference range 130 g/L), and platelet count of 39 × 10^9^/L (lower limit of normal reference range 100 g/L). Computed tomography enhanced scan of the upper abdomen demonstrated evidence of liver cirrhosis, splenomegaly, esophageal varices, massive ascites, and gallstone (Fig. 1A). The preoperative Child–Pugh score and MELD score were respectively calculated at 13 and 41 (Table 1).

**Table 1:**
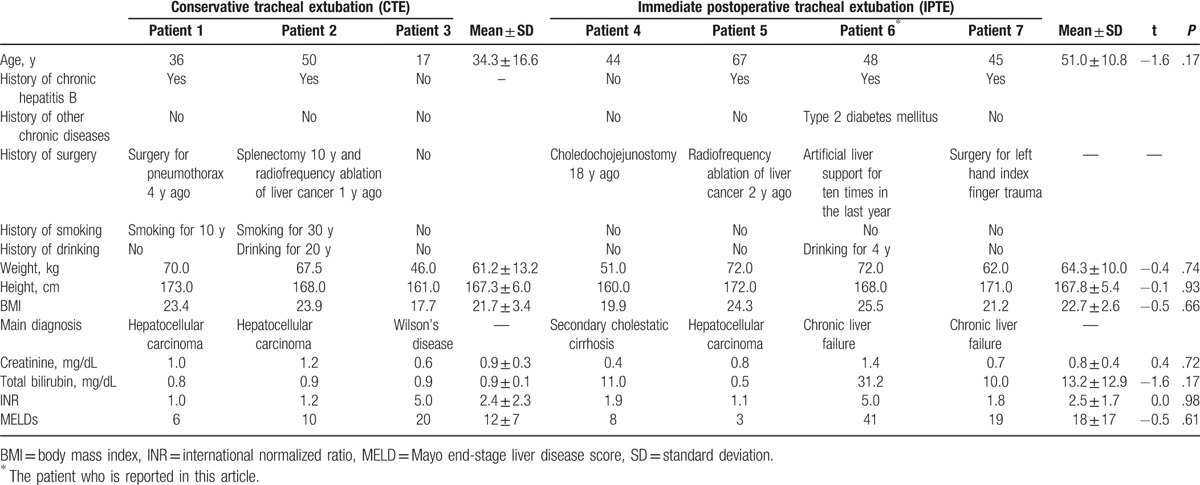
Preoperative variables of patients undergoing liver transplantation in the same month.

Procurement of the piggyback liver transplantation was performed using standard techniques.^[5,6]^ The operation, with a blood loss of 1000 mL and total blood transfusion of 2950 mL (autologous blood of 500 mL, red blood cell suspension of 8 U, and plasma 850 mL), went smoothly and lasted a total of 6.3 hours. The gross appearance of the explanted liver confirmed the computed tomography scan findings of multinodular cirrhosis (Fig. 1B and Table 2).

**Table 2:**
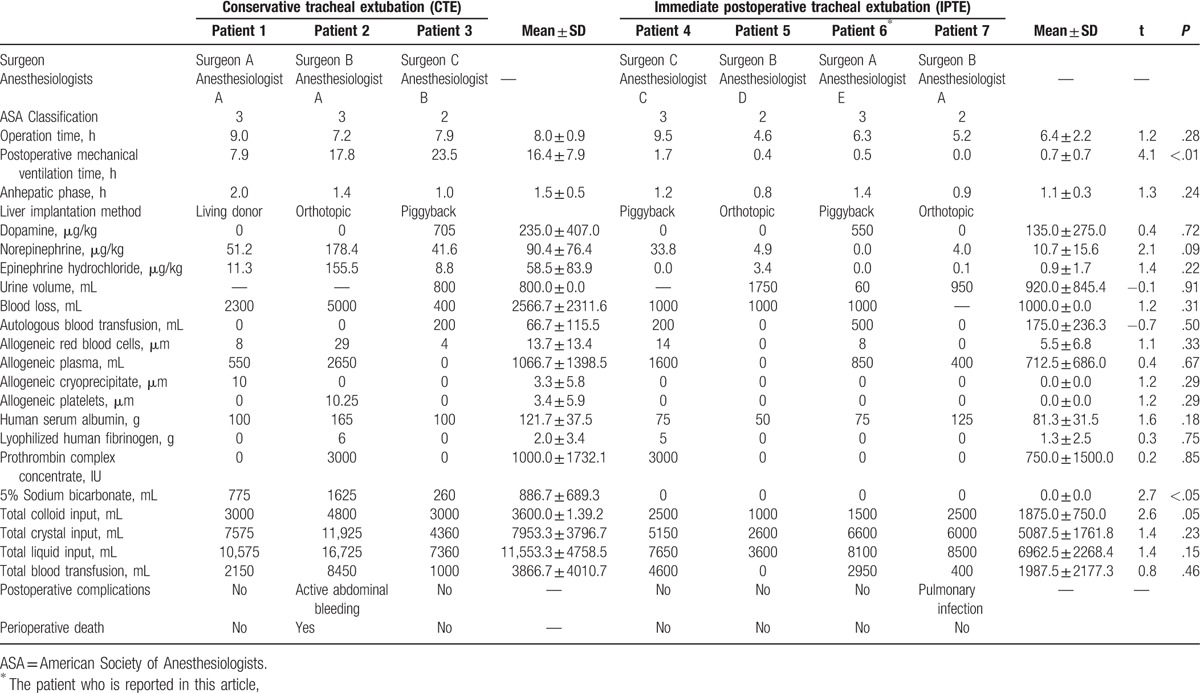
Intraoperative variables and postoperative complications of patients undergoing liver transplantation in the same month.

The anesthesia program included induction with intravenous propofol 2 to 3 mg/kg, fentanyl 0.4 μg/kg, midazolam 2 mg, penehyclidine hydrochloride 0.1 mg/kg and rocuronium 1 mg/kg, and maintenance with 2% sevoflorane in a 50% air/oxygen low-flow respiratory mixture, remifentanil (0.1–0.2 μg/kg/min), and cisatracurium (10 mg/hour). Hemodynamic monitoring included invasive systemic arterial pressure and the use of a central venous catheter. The 1% ropivacaine was subcutaneously injected along the incision when suturing the skin.

At the end of the operation, the patient's hemodynamic stability was determined by the attending anesthetist in the operating room and then tracheal extubation was carried out according to the standardized and the universally accepted criteria (patient awake, ability to lift the head and swallow, and good oxygenation). Subsequently, the patient was admitted to the intensive care unit (ICU).

The patient made an excellent postoperative recovery and transferred to the surgical ward from the ICU 2 days later. He was discharged on the day 10, with a lower hospital cost compared to the average for liver transplantation.

## 3 Discussion

To the best of our knowledge, this case is the first to report IPTE in a liver transplant recipient with encephalopathy and so high MELD score up to 41. In fact, IPTE once relating to fast tracking which emphasizes efficient use of resources^[7]^ is now one potential subtopic of currently the most popular surgical theme called ERAS^[8]^ which our liver transplantation center is committed to practice at present.

Although the early extubation was introduced to fast tracking in patients after coronary artery bypass grafting in 1980,^[9]^ the similar practice in liver transplant recipients was conducted by Mandell et al^[2]^ 17 years later. Theoretically, positive pressure ventilation and positive end expiratory pressure can reduce liver blood flow and are especially maligned in the context of compromised immune and high cardiac indices during liver transplantation. In the last 15 years, a series of studies has been demonstrated that IPTE in the operating room could be successfully performed in a large fraction of patients (60%–80%) without an increased risk of subsequent reintubation.^[10–12]^

According to the available literature,^[1,7,10]^ benefits of such an approach include: higher quality of care, shorter ICU and hospital length of stay, and less total treatment costs. In line with these reports, our patient's postoperative course was uneventful, and he was discharged home on day 10. Also his hospital cost was below average level for liver transplantation.

Immediate extubation following liver transplantation has been existed for decades, however, considering possible failure of IPTE, which patients are more suitable for IPTE or what predict IPTE is still a question. In Europe, Biancofiore et al's study^[1]^ showed that only an MELD score <11 could predict the successful IPTE with a receiver operator characteristic area under the curve of 0.61, but the pretransplant Child–Pugh score did not. Perkins^[13]^ did not think this criterion would be suitable for liver transplantation in the United States, which were performed mainly for patients with a MELD score >16. A further statistical analysis in a similar patient population demonstrated that the only factors associated with the failure to IPTE were encephalopathy and increased body mass index >34.^[3]^ Different from the above studies, a recent study showed the patient's initial hemoglobin concentration, the number of packed red blood cells and fresh frozen plasma transfused during surgery, and pain control by application of a thoracic epidural catheter were only 3 independent predictors of IPTE.^[12]^ Interestingly, our case with an MELD score of 41, encephalopathy and massive intraoperative blood transfusion failed to meet the above criteria for IPTE does show a good recovery result.

In order to explore decisive factors of IPTE in this case, we did a retrospective analysis which involved another 6 cases in our center during the same period. We did a total of 9 liver transplantations in September including 7 adult and 2 pediatric cases. Of the 7 adult cases, 3 underwent conservative tracheal extubation (CTE) with an average value of 16.4 hours for postoperative mechanical ventilation and 4 underwent IPTE with an average time of 0.7 hours (Table 2). We compared the 2 groups of patients from preoperative variables, intraoperative variables and postoperative complications, together with the intraoperative intraoperative arterial blood gas analysis results (Tables 1–3). We found there was no significant difference between the 2 groups for most of the variables; however, the intraoperative transfusion volume of 5% sodium bicarbonate and PO_2_ at the end of surgery differed in the 2 groups (Tables 2, 3). In addition, the intraoperative total colloid input volume also showed a marginal difference between CTE and IPTE. It is also noteworthy that our evidence is not definitive due to the limited number of cases included in our analysis and more large sample studies are required in the future. Nevertheless, a more detailed analysis revealed that all the patients in the IPTE had no smoking history and this case we reported in the above did not use any vasoconstrictor including norepinephrine and epinephrine hydrochloride, which indicated a good lung function and a stable intraoperative hemodynamic should be important for this challenging practice.

**Table 3:**
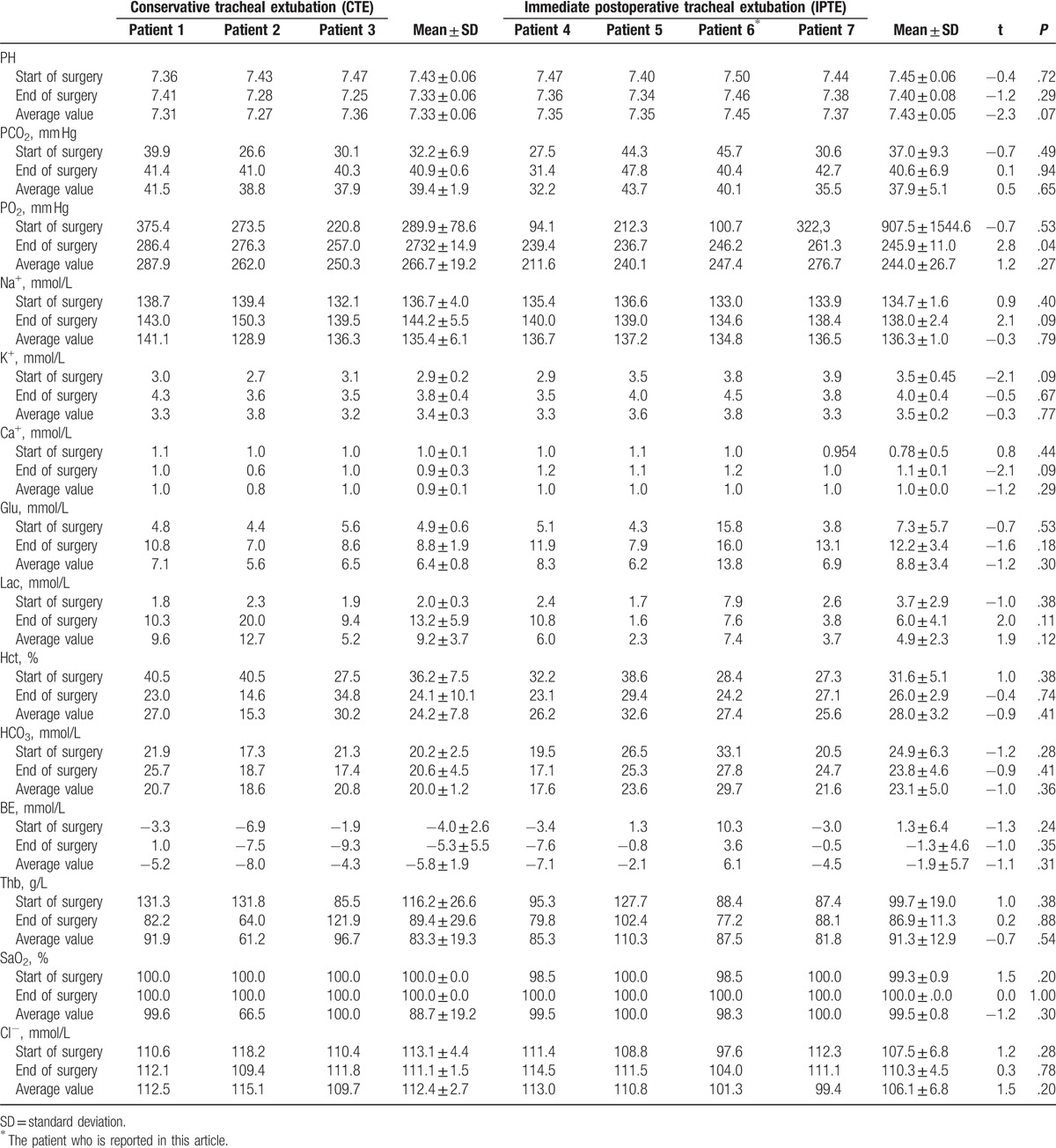
Intraoperative arterial blood gas analysis results of patients undergoing liver transplantation in the same month.

Our case suggests even for recipients with encephalopathy and a high MELD score, successful IPTE in liver transplantation still exists in certain cases. Furthermore, a larger series that provides who to select for early extubation and the pros/cons would be useful in the practice of ERAS for liver transplantation.

## References

[1] Biancofiore G Bindi ML Romanelli AM Fast track in liver transplantation: 5 years’ experience. Eur J Anaesthesiol 2005;22:584–90. 16119594 10.1017/s0265021505000980

[2] Mandell MS Lockrem J Kelley SD Immediate tracheal extubation after liver transplantation: experience of two transplant centers. Anesth Analg 1997;84:249–53. 9024010 10.1097/00000539-199702000-00003

[3] Mandell MS Lezotte D Kam I Reduced use of intensive care after liver transplantation: patient attributes that determine early transfer to surgical wards. Liver Transpl 2002;8:682–7. 12149760 10.1053/jlts.2002.34380

[4] Skurzak S Stratta C Schellino MM Extubation score in the operating room after liver transplantation. Acta Anaesthesiol Scand 2010;54:970–8. 20626358 10.1111/j.1399-6576.2010.02274.x

[5] Starzl TE Marchioro TL Vonkaulla KN Homotransplantation of the liver in humans. Surg Gynecol Obstet 1963;117:659–76. 14100514 PMC2634660

[6] Gerber DA Passannante A Zacks S Modified piggyback technique for adult orthotopic liver transplantation. J Am Coll Surg 2000;191:585–9. 11085741 10.1016/s1072-7515(00)00702-x

[7] Plevak DJ Torsher LC Fast tracking in liver transplantation. Liver Transpl Surg 1997;3:447–8. 9346779 10.1002/lt.500030419

[8] Steenhagen E Enhanced recovery after surgery: it's time to change practice!. Nutr Clin Pract 2016;31:18–29. 26703956 10.1177/0884533615622640

[9] Quasha AL Loeber N Feeley TW Postoperative respiratory care: a controlled trial of early and late extubation following coronary-artery bypass grafting. Anesthesiology 1980;52:135–41. 6986104

[10] Glanemann M Busch T Neuhaus P Fast tracking in liver transplantation. Immediate postoperative tracheal extubation: feasibility and clinical impact. Swiss Med Wkly 2007;137:187–91. 17525870 10.4414/smw.2007.11681

[11] Aniskevich S Pai SL Fast track anesthesia for liver transplantation: review of the current practice. World J Hepatol 2015;7:2303–8. 26380654 10.4254/wjh.v7.i20.2303 PMC4568490

[12] Blaszczyk B Wronska B Klukowski M Factors affecting breathing capacity and early tracheal extubation after liver transplantation: analysis of 506 cases. Transplant Proc 2016;48:1692–6. 27496473 10.1016/j.transproceed.2016.01.053

[13] Perkins JD Immediate tracheal extubation following liver transplantation. Liver Transpl 2006;12:883–4. 16628681 10.1002/lt.20774

